# Acute-Onset Dystonia Following Ashwagandha Supplementation (Withania somnifera): A Case Report

**DOI:** 10.7759/cureus.89104

**Published:** 2025-07-31

**Authors:** Misbah Fazlani, Pushparaja Shetty, Ahmer Longi, Sidra Akram, Saima Nawab

**Affiliations:** 1 Internal Medicine, Mediclinic Welcare Hospital, Dubai, ARE; 2 Neurology, Mediclinic Welcare Hospital, Dubai, ARE

**Keywords:** ashwagandha, case report, dystonia, herbal supplement, movement disorder, oculogyric crisis

## Abstract

Ashwagandha (Withania somnifera) is a widely used herbal supplement with established adaptogenic and neuroprotective properties. Although generally considered safe, rare adverse neurological effects may occur. We present the case of a previously healthy adult male who developed acute-onset dystonia following the initiation of Ashwagandha supplementation. The patient exhibited sustained involuntary muscle contractions and abnormal posturing shortly after commencing the supplement. Extensive diagnostic evaluation failed to identify an alternative etiology. Discontinuation of Ashwagandha and initiation of symptomatic treatment led to the resolution of symptoms. This case underscores the importance of considering herbal supplements as potential contributors to neurological presentations.

## Introduction

Ashwagandha (Withania somnifera) is a medicinal herb widely used in traditional Ayurvedic medicine. It is known for its adaptogenic properties and is commonly used in Southeast Asia to manage symptoms of anxiety and stress. In recent decades, growing scientific interest has explored its potential therapeutic benefits across a range of neuropsychiatric conditions, including anxiety, depression, and insomnia. These effects are thought to be mediated by modulation of the hypothalamic-pituitary-adrenal (HPA) axis, regulation of cortisol secretion, and enhancement of gamma-aminobutyric acid (GABA) receptor activity [1-4].

Preclinical studies support Ashwagandha’s anxiolytic and antidepressant properties through neuroprotective and antioxidant mechanisms [1,2]. It is often referred to as the “Indian ginseng” due to its broad-spectrum adaptogenic and neuropsychiatric effects [2,5]. Clinically, a randomized, double-blind, placebo-controlled trial by Chandrasekhar et al. demonstrated that a high-concentration extract of Ashwagandha root significantly reduced perceived stress and anxiety in adults, with an excellent safety profile [3]. Systematic reviews have further supported its role in improving sleep quality and reducing neuropsychiatric symptoms across diverse populations [4]. These findings contribute to its growing acceptance as a complementary therapy in modern medical practice.

Although Ashwagandha is generally considered safe in controlled settings, emerging reports suggest potential neuropsychiatric side effects, particularly when used alongside other psychoactive medications [6]. These observations underscore the importance of pharmacovigilance, even with herbal supplements commonly perceived as benign. Clinicians may overlook these risks, especially when patients use over-the-counter Ayurvedic products without disclosing them during medical consultations.

In this context, we present the case of a previously healthy adult male who developed acute-onset dystonia temporally associated with Ashwagandha supplementation. This report highlights the need for clinician awareness of potential herb-drug interactions and reinforces the importance of monitoring for adverse neurological effects, even in supplements with an established reputation for safety.

## Case presentation

A 40-year-old Asian male presented to the neurology outpatient clinic with a two-week history of intermittent abnormal neck movements, sustained conjugate ocular deviation to the left, and unsteady gait. His symptoms were characterized by painful involuntary neck muscle contractions, transient speech difficulty (aphonia), and episodes of sustained eye deviation. According to his wife, he also experienced intermittent delayed responsiveness and several near-falls, veering unpredictably while walking.

Symptom onset occurred approximately one week after initiating daily self-administration of a commercially available Ashwagandha (Withania somnifera) supplement. He had taken it for a total of 25 days prior to presentation, primarily to alleviate stress and improve sleep. He denied recent infections, fever, head trauma, seizures, alcohol or recreational drug use, or exposure to known neurotoxins. There was no history of psychiatric illness, and he was not on any chronic prescription medications except for intermittent use of diazepam 2.5 mg twice daily for anxiety, which he had started before initiating Ashwagandha and had not recently adjusted.

His past medical history was significant for hyperlipidemia, for which he was not actively treated. Family history was notable for premature coronary artery disease. There was no personal or family history of movement disorders or neuropsychiatric conditions.

Examination

On physical examination, the patient was alert and oriented. Neurological evaluation during spontaneous episodes revealed dystonic posturing, including sustained ocular deviation to the left (oculogyric crisis) and cervical dystonia. Between episodes, cranial nerve examination was normal, with intact motor, sensory, and cerebellar systems. Deep tendon reflexes were normal and symmetric. Gait assessment revealed cautious ambulation with mild instability but no frank ataxia. Cardiovascular and other systemic examinations were unremarkable.

Investigations

**Figure 1:**
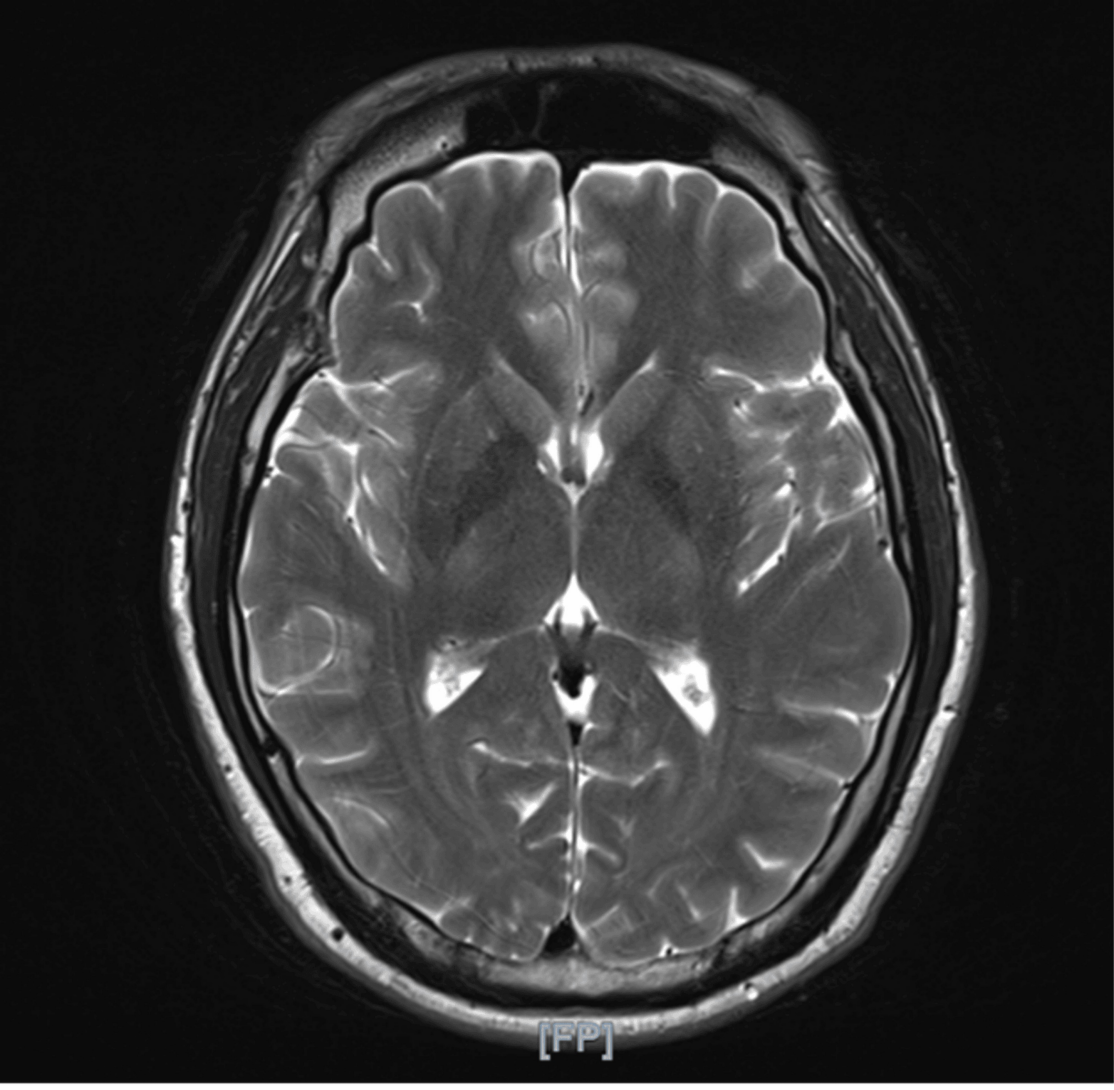
Axial T2-weighted MRI of the brain demonstrating no acute pathology. No evidence of stroke, demyelination, or basal ganglia lesions.

Routine laboratory investigations - including complete blood count, serum electrolytes, renal and liver function tests, thyroid function tests, and inflammatory markers - were all within normal limits (Table 1). Brain magnetic resonance imaging (MRI) with contrast demonstrated no structural abnormalities or signs of stroke, demyelination, or basal ganglia involvement (Figure 1). Electroencephalography (EEG) revealed normal background activity without epileptiform discharges or focal slowing.

**Table 1:** Laboratory investigations revealed values within normal reference ranges, including complete blood count, electrolytes, liver and renal function tests, inflammatory markers, and autoimmune panels. These normal results effectively ruled out systemic infections, metabolic imbalances, and autoimmune conditions as contributors to the patient’s dystonia. ESR: Erythrocyte sedimentation rate; eGFR: estimated glomerular filtration rate; CRP: C-reactive protein; ALT: Alanine transaminase; ALP: Alkaline phosphatase; AST: Aspartate transaminase; TSH: Thyroid-stimulating hormone; PT: Prothrombin Time; INR: International normalized ratio; aPTT: activated partial thromboplastin time.

Test	Result	Reference Range	Unit
ESR	6	2 - 30	mm/hr
Calcium (Ca)	2.30	2.15 - 2.5	mmol/L
Ceruloplasmin	0.196	0.15 - 0.3	g/L
Copper	89	70.0 - 140.0	µg/dL
Creatinine (Cr)	77	80 - 115	µmol/L
eGFR	110	> 60	mL/min/1.73 m^2^
CRP	1.4	0.0 - 5.0	mg/L
Magnesium (Mg)	0.853	0.636 - 1.07	mmol/L
Vitamin B12	361	145 - 569	pmol/L
Phosphate (PO4)	1.25	0.81 - 1.45	mmol/L
Total Bilirubin (Tb)	5.85	< 21.0	µmol/L
Direct Bilirubin	2.77	≤ 7.0	µmol/L
ALT	22	< 50	U/L
ALP	22	40 - 129	U/L
AST	24	< 50	U/L
Sodium (Na)	140	136 - 145	mmol/L
Potassium (K)	4.0	3.5 - 5.1	mmol/L
Chloride (Cl)	101	98 - 107	mmol/L
TSH	1.62	0.27 - 4.20	µIU/mL
Hemoglobin (Hb)	14	3.0 - 17.5	g/dL
White Blood Cells (WBC)	10	4 - 11	10^3^/µL
Platelets (Plt)	253	150 - 450	10^3^/µL
PT	13	11.7 - 15.3	seconds
INR	0.92	0.80 - 1.20	Ratio
aPTT	33	28.6 - 40.0	seconds

Autoimmune and connective tissue workup - including antinuclear antibody (ANA) profile, rheumatoid factor (RF), and autoimmune encephalitis panel - was negative. Blood cultures remained sterile throughout hospitalization.

Diagnosis and management

A comprehensive differential diagnosis was considered, including primary dystonia, structural brain lesions, autoimmune encephalitis, metabolic or infectious causes, and drug-induced movement disorders. Given the absence of alternative etiologies, the temporal relationship with Ashwagandha use, and the known pharmacological properties of the herb, a diagnosis of acute dystonic reaction - likely precipitated by Ashwagandha - was made. Literature and pharmacovigilance data suggest that Ashwagandha may influence dopaminergic and cholinergic neurotransmission, potentially triggering dystonia in susceptible individuals, especially when used alongside psychoactive medications such as benzodiazepines.

The patient was started on procyclidine (Kemadrin) 5 mg three times daily, a single dose of promethazine 25 mg, and continuation of diazepam 2.5 mg. He demonstrated marked improvement within 24 hours, with complete resolution of dystonic symptoms.

Outcome and follow-up

At one-week follow-up, the patient remained asymptomatic. His gait, speech, and eye movements were entirely normal on examination, and he denied any recurrence of dystonic episodes. He reported discontinuation of Ashwagandha and had not initiated any other supplements. Neurological examination remained unremarkable, and no further treatment was necessary.

## Discussion

Ashwagandha (Withania somnifera) is a widely used adaptogenic herb recognized for its anxiolytic, antidepressant, and neuroprotective properties, supported by both preclinical and clinical evidence [1-4]. These effects are primarily attributed to its bioactive constituents - namely withanolides and alkaloids - which have been shown to modulate gamma-aminobutyric acid (GABA) receptor activity, reduce cortisol levels, and exert antioxidative and anti-inflammatory actions [1,2,4]. For example, Bhattacharya et al. demonstrated anxiolytic and antidepressant effects in animal models, attributed to GABAergic and stress-attenuating pathways [1], while Kulkarni and Dhir described Ashwagandha’s broad-spectrum neuropharmacological effects, including its action on serotonergic and cholinergic systems [2].

Chandrasekhar et al. conducted a high-quality randomized controlled trial that confirmed Ashwagandha's efficacy in reducing stress and anxiety in adults, with a strong safety profile in controlled settings [3]. Systematic reviews have also supported its benefit across various neuropsychiatric conditions, including insomnia and depression [4]. However, these trials often exclude individuals with psychiatric comorbidities or concurrent use of psychoactive medications, limiting their applicability to real-world patient populations [4].

This case highlights a rare but clinically significant adverse neurological reaction - acute dystonia - associated with Ashwagandha use. Dystonia is a movement disorder characterized by sustained or intermittent involuntary muscle contractions leading to abnormal postures or repetitive movements. While most commonly linked to dopamine receptor blockade, imbalances in GABAergic, serotonergic, and cholinergic neurotransmission can also play a role in its pathogenesis.

Preclinical studies suggest that Ashwagandha enhances GABAergic tone, likely via GABA-A receptor modulation [1,2], which may underlie its calming effects. However, enhanced GABAergic or serotonergic activity - especially when combined with CNS-active agents like benzodiazepines - may disrupt the balance of neurotransmitter systems and trigger movement disorders in susceptible individuals. In this case, the patient was taking a standardized Ashwagandha extract (600 mg/day, ≥5% withanolides) for 25 days along with intermittent diazepam use. A comprehensive diagnostic workup ruled out alternative etiologies, and symptoms resolved rapidly upon discontinuation of Ashwagandha and initiation of anticholinergic therapy.

While direct antidopaminergic activity has not been demonstrated, Ashwagandha’s influence on serotonergic and possibly cholinergic pathways could indirectly modulate dopaminergic tone [2,5], contributing to movement disorders in predisposed individuals. Sharma and Singh’s recent review of pharmacovigilance data and case reports underscores this concern, documenting neurological adverse effects - including dystonia - associated with herbal supplements like Ashwagandha, particularly in the context of polypharmacy or unsupervised use [6].

This case reinforces several key clinical messages. First, clinicians should obtain a detailed history of herbal supplement use when evaluating unexplained neuropsychiatric or movement symptoms. Second, while Ashwagandha is generally well-tolerated, individual variability, formulation differences, and potential herb-drug interactions can lead to rare but serious adverse events. Finally, increased pharmacovigilance and clinician awareness are essential as complementary and alternative medicines become more widely integrated into clinical practice.

## Conclusions

Ashwagandha is widely used for its adaptogenic and neuroprotective effects; however, this case illustrates that it can, in rare instances, induce acute dystonia likely via modulation of GABAergic and serotonergic neurotransmission. Clinicians should maintain awareness of potential adverse neurological effects associated with herbal supplements and carefully evaluate supplement use during patient assessment. Prompt recognition and discontinuation of Ashwagandha, alongside symptomatic treatment, can lead to full recovery. Increased pharmacovigilance and research are needed to elucidate mechanisms, risk factors, and to guide clinical management of such rare adverse events.
